# Control of lupus nephritis by changes of gut microbiota

**DOI:** 10.1186/s40168-017-0300-8

**Published:** 2017-07-11

**Authors:** Qinghui Mu, Husen Zhang, Xiaofeng Liao, Kaisen Lin, Hualan Liu, Michael R. Edwards, S. Ansar Ahmed, Ruoxi Yuan, Liwu Li, Thomas E. Cecere, David B. Branson, Jay L. Kirby, Poorna Goswami, Caroline M. Leeth, Kaitlin A. Read, Kenneth J. Oestreich, Miranda D. Vieson, Christopher M. Reilly, Xin M. Luo

**Affiliations:** 1 Department of Biomedical Sciences and Pathobiology, Virginia-Maryland College of Veterinary Medicine, Virginia Tech, Blacksburg, VA USA; 2 Department of Civil and Environmental Engineering, Virginia Tech, Blacksburg, VA USA; 3 Department of Biological Sciences, Virginia Tech, Blacksburg, VA USA; 4 Department of Animal and Poultry Sciences, Virginia Tech, Blacksburg, VA USA; 5 Virginia Tech Carilion Research Institute and School of Medicine, Roanoke, VA USA; 6 Edward Via College of Osteopathic Medicine, Blacksburg, VA USA; 7 Present Address: Cancer and Inflammation Program, Center for Cancer Research, National Cancer Institute, Bethesda, MD 20892 USA; 8 Present Address: Lawrence Berkeley National Laboratory, Berkeley, CA 94720 USA

**Keywords:** Gut microbiota, Lupus, Leaky gut, Autoimmunity

## Abstract

**Background:**

Systemic lupus erythematosus, characterized by persistent inflammation, is a complex autoimmune disorder with no known cure. Immunosuppressants used in treatment put patients at a higher risk of infections. New knowledge of disease modulators, such as symbiotic bacteria, can enable fine-tuning of parts of the immune system, rather than suppressing it altogether.

**Results:**

Dysbiosis of gut microbiota promotes autoimmune disorders that damage extraintestinal organs. Here we report a role of gut microbiota in the pathogenesis of renal dysfunction in lupus. Using a classical model of lupus nephritis, MRL/*lpr*, we found a marked depletion of *Lactobacillales* in the gut microbiota. Increasing *Lactobacillales* in the gut improved renal function of these mice and prolonged their survival. We used a mixture of 5 *Lactobacillus* strains (*Lactobacillus oris*, *Lactobacillus rhamnosus*, *Lactobacillus reuteri*, *Lactobacillus johnsonii*, and *Lactobacillus gasseri*), but *L. reuteri* and an uncultured *Lactobacillus* sp. accounted for most of the observed effects. Further studies revealed that MRL/*lpr* mice possessed a “leaky” gut, which was reversed by increased *Lactobacillus* colonization. *Lactobacillus* treatment contributed to an anti-inflammatory environment by decreasing IL-6 and increasing IL-10 production in the gut. In the circulation, *Lactobacillus* treatment increased IL-10 and decreased IgG2a that is considered to be a major immune deposit in the kidney of MRL/*lpr* mice. Inside the kidney, *Lactobacillus* treatment also skewed the Treg-Th17 balance towards a Treg phenotype. These beneficial effects were present in female and castrated male mice, but not in intact males, suggesting that the gut microbiota controls lupus nephritis in a sex hormone-dependent manner.

**Conclusions:**

This work demonstrates essential mechanisms on how changes of the gut microbiota regulate lupus-associated immune responses in mice. Future studies are warranted to determine if these results can be replicated in human subjects.

**Electronic supplementary material:**

The online version of this article (doi:10.1186/s40168-017-0300-8) contains supplementary material, which is available to authorized users.

## Background

Perturbation of gut microbiota is known to promote autoimmune disorders that include inflammatory bowel disease, type 1 diabetes, rheumatoid arthritis, and multiple sclerosis. However, little is known on the role of gut microbiota in systemic lupus erythematosus (SLE). SLE is a very complex autoimmune disorder with no known cure. It is characterized by severe and persistent inflammation that damages multiple organs, including the skin, kidney, lung, joint, heart, and brain [1]. The prevalence ranges from 20 to 200 cases per 100,000 persons, with higher prevalence for people of African, Hispanic, or Asian ancestry. Although the disease affects both males and females, women of childbearing age are diagnosed 9 times more often than men. African-American women suffer from more severe symptoms and a higher mortality rate. More than half of SLE patients suffer from kidney inflammation, or lupus nephritis (LN), which is the leading cause of mortality by SLE. Current treatments for LN are primarily nonselective immunosuppressants [2]. While immunosuppression can effectively treat symptoms, unwanted side effects are a major cause of concern. Patients taking long-term immunosuppressants are prone to higher incidence of and more severe infections [3]. Therefore, there is an imperative need for new treatment strategies against LN. To accomplish this task, a better understanding of disease pathogenesis is required.

Current knowledge on the relationship between gut microbiota and SLE is limited [4]. In human SLE, a recent cross-sectional study showed dysregulated fecal microbiota of SLE individuals with a lower *Firmicutes* to *Bacteroidetes* ratio [5] that is consistent with gut dysbiosis observed in other autoimmune conditions [6, 7]. In mice, it has been reported that the lupus-prone MRL/Mp-*Fas* ^*lpr*^ (*lpr*) mouse model exhibits similar disease manifestations under specific pathogen-free and germ-free conditions [8]. This suggests that complete removal of microbiota does not affect disease progression in these mice. The same phenomenon was observed in the pristane-induced lupus model [9]. However, completely depleting the microbiota might have neutralized the respective effects of “good” and “bad” microbes. Studies on germ-free New Zealand black mice showed mixed results, with less renal disease but more anti-nuclear antibodies [10–12]. Our research team has recently described the dynamics of fecal/colonic microbiota in *lpr* mice that suggests a critical role of gut microbiota on lupus pathogenesis [13]. However, whether the change of gut microbiota is a driving force in SLE, or merely a result of disease status, remains unclear.

Here we show that intestinal permeability is increased in female *lpr* mice preceding the onset of kidney disease (i.e., a “leaky” gut) and that increasing gut colonization of *Lactobacillales* restores the mucosal barrier function and reduced kidney pathology. Such change in gut microbiota promotes an anti-inflammatory environment in the gut, suppressing expression of IL-6 in the mesenteric lymph node (MLN) while increasing the levels of IL-10 in circulation and periphery. In addition, the production and renal deposition of pathogenic IgG2a is repressed with increased *Lactobacillales*, suggesting a potential mechanism for the reduced kidney pathology. Moreover, we show that *Lactobacillus* spp. rebalances T cell subsets in the kidney, increasing regulatory T (Treg) cells and suppressing pathogenic T-helper (Th) 17 cells. This suggests another potential mechanism by which gut microbiota can modulate renal function. Interestingly, the effects of *Lactobacillus* spp. are only present in female and castrated male *lpr* mice, but not in intact males, indicating a role for sex hormones in the regulatory function of gut microbiota on lupus disease. Taken together, our results suggest that the presence of *Lactobacillus* spp. in the gut can attenuate kidney inflammation in lupus-prone mice in a sex hormone-dependent manner.

## Results

### *Lactobacillus* spp. attenuate LN

When comparing the bacterial composition in the gut microbiota of lupus-prone *lpr* mice vs. MRL control mice, we found that female *lpr* mice had a significantly lower abundance of *Lactobacillales* in the gut microbiota than MRL controls at 5 weeks of age and prior to the onset of lupus-like disease (Additional file 1: Figure S1A). However, it was unclear whether the change was a cause or result of disease initiation. Therefore, we performed reciprocal cecal microbiota transplantation experiments from MRL to *lpr* mice (Additional file 1: Figure S1B) and vice versa. While the disease in MRL mice did not change after the transfer of cecal content from *lpr* mice (data not shown), MRL-to-*lpr* cecal transplantation led to significantly reduced production of autoantibodies against double-stranded (ds) DNA from the lower gastrointestinal tract (Additional file 1: Figure S1C). Since the gut microbiota of young MRL mice contained a higher abundance of *Lactobacillales* than *lpr* mice, we sought to determine if the decrease in disease could be due to the elevated *Lactobacillales* in *lpr* mice that were transferred from MRL mice upon cecal transplantation. Indeed, *lpr* mice receiving MRL cecal content had more abundant *Lactobacillales* in the gut microbiota than untreated controls (Additional file 1: Figure S1D), suggesting a positive correlation between a higher abundance of gut-colonized *Lactobacillales* and improved lupus symptoms.

**Fig. 1:**
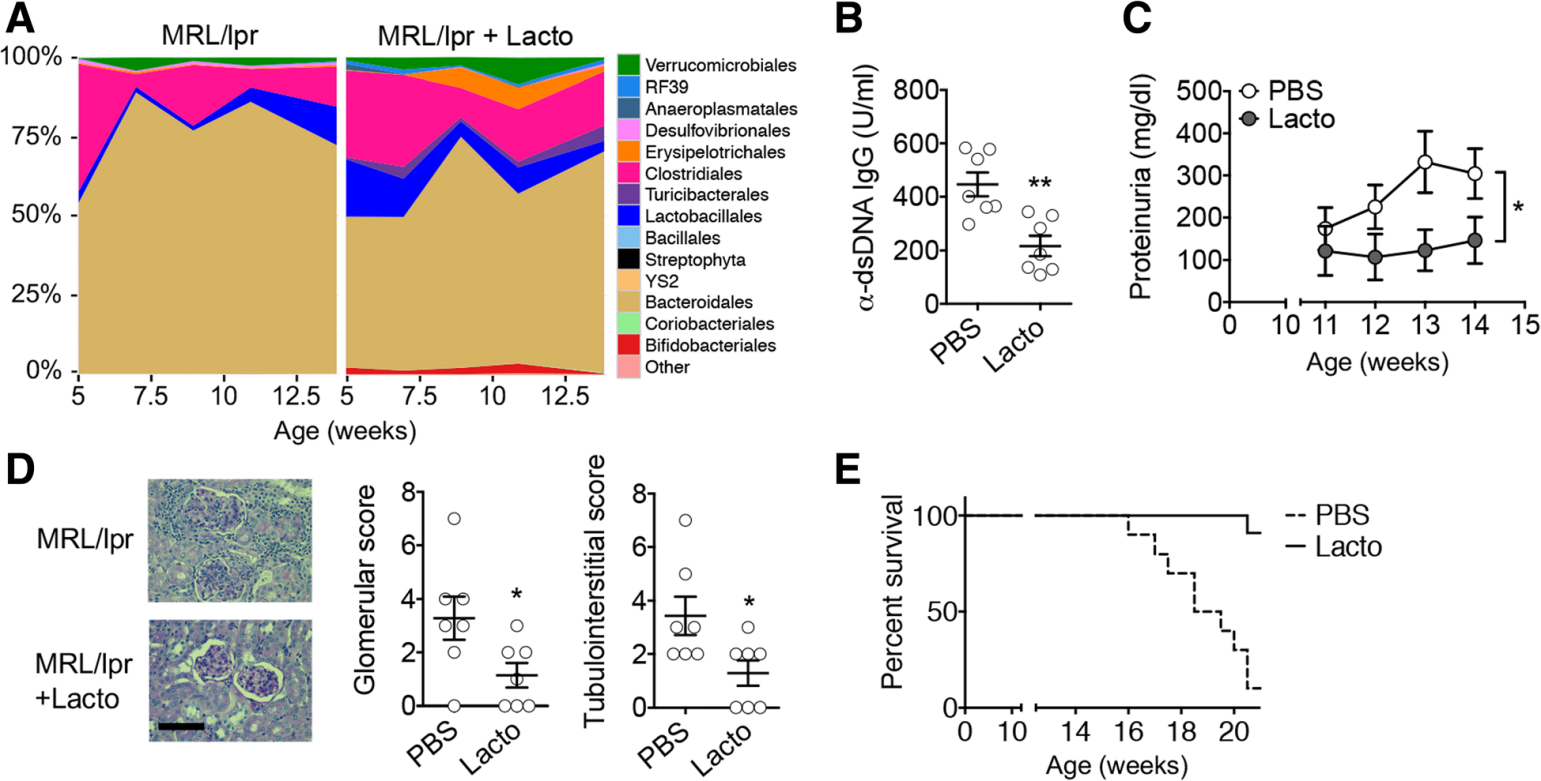
*Lactobacillus* spp. protect female *lpr* mice from LN. **a** Time-dependent changes of fecal microbiota upon PBS or *Lactobacillus* (*Lacto*) treatment (*n* = 4 mice per group). **b** Level of anti-dsDNA IgG in the blood of 10-week-old mice (*n* = 7 mice per group; **P < 0.01). **c** Level of proteinuria over time (*n* = 7 mice per group; paired *t* test; *P < 0.05). **d** Renal histopathology at 14 weeks of age (*n* = 7 mice per group; chi-square test; *P < 0.05). *Left*: PAS-stained kidney sections; *bar* equals 100 μm. **e** Survival rate (*n* = 10 mice per group; chi-square test; ****P < 0.0001)

The bacterial order *Lactobacillales* includes *Lactobacillus* spp. that are known as beneficial bacteria. We thus examined the effect of these beneficial bacteria on *lpr* mice by directly inoculating freshly cultured *Lactobacillus* isolates (Additional file 1: Figure S1E). We used a mixture of 5 *Lactobacillus* strains—*Lactobacillus oris*, *Lactobacillus rhamnosus*, *Lactobacillus reuteri*, *Lactobacillus johnsonii*, and *Lactobacillus gasseri*. Different *Lactobacillus* strains have been reported to exert different immunological functions [14, 15]. Among the 5 strains, all except *L. oris* are known to colonize the gut. To improve engraftment of *Lactobacillus* spp., we pre-treated the mice with ampicillin, neomycin, vancomycin, and metronidazole for 2 days, followed by 2 days of resting to allow for excretion of the antibiotics prior to *Lactobacillus* treatment. The brief antibiotic treatment at the time of weaning did not change the disease severity (Additional file 1: Figures S1F and S1G). We found that weekly gavages of *Lactobacillus* spp. significantly increased the relative abundance of *Lactobacillales* in the gut microbiota at weeks 5 and 7 (Fig. 1a and Additional file 2: Table S1), significantly reduced the level of autoantibodies in the circulation (Fig. 1b), and significantly decreased proteinuria (Fig. 1c) and renal pathology scores (Fig. 1d). The spleen and MLN weights were not changed (Additional file 1: Figure S1H). Importantly, *Lactobacillus* treatment significantly increased the survival of female *lpr* mice (Fig. 1e). It is noteworthy that *Lactobacillus* treatment was given starting from 3 weeks of age and before disease establishment. When given after the onset of lupus disease, *Lactobacillus* treatment had a trend to reduce lupus disease, but the difference was not statistically significant (data not shown). These results suggest that the introduction of more “good” bacteria in the gut microbiota—in this case, *Lactobacillus* spp.—may be able to prevent disease progression in lupus-prone mice. This supports the notion that gut microbiota can directly control LN. How the increase of *Lactobacilli* in the gut affects disease pathogenesis in the kidney, which is extraintestinal, was unknown. Therefore, we next sought to identify potential “messengers” that transduced the disease-modulating signal from the gut to the kidney.

### A “leaky” gut in lupus-prone mice

**Fig. 2:**
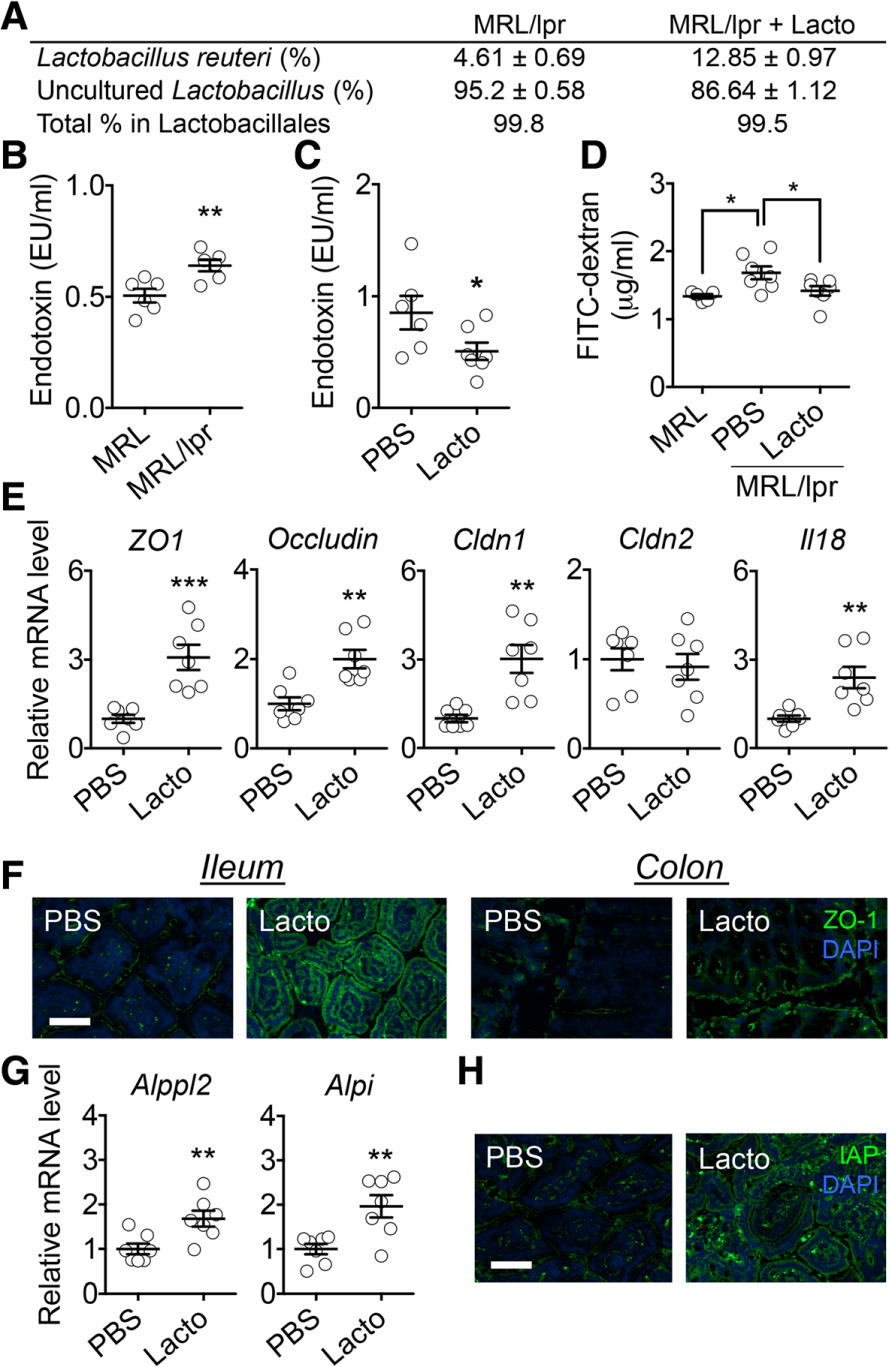
*Lactobacillus* spp. restore gut mucosal barrier function in female *lpr* mice. **a** Percentage of *Lactobacillus* strains in the order *Lactobacillales* (*n* = 4 per group). **b** Level of endotoxin in the blood of 6-week-old *lpr* mice (*n* = 6 mice per group; **P < 0.01). **c** Level of endotoxin in the blood of 10-week-old *lpr* mice with or without *Lactobacillus* treatment (*n* = 6 or 7 mice per group; *P < 0.05). **d** Level of FITC-dextran diffused to the blood (*n* = 5 or 7 mice per group; *P < 0.05). **e** Transcript levels of tight junction proteins and IL-18 in intestinal epithelial cells of 14-week-old *lpr* mice (*n* = 7 mice per group; **P < 0.01, ***P < 0.005). **f** Immunohistochemical stains of ZO-1 (*green*) in the ileum or colon. Nuclear stain (DAPI) is shown in *blue. Bar* equals 75 μm. **g** Transcript levels of IAP genes in the epithelium (*n* = 7 mice per group; **P < 0.01). **h** Immunohistochemical stains of IAP (*green*) in the ileum. *Bar* equals 75 μm

While 5 *Lactobacillus* strains were inoculated, we found by using 16S ribosomal RNA gene sequencing that, unexpectedly, two bacterial species accounted for >99% of the order *Lactobacillales* regardless of treatment status. The species were *L. reuteri* and an uncultured *Lactobacillus* sp. (Fig. 2a). The same phenomenon was observed for MRL mice (data not shown). This suggests that *L. reuteri* and the uncultured *Lactobacillus* sp. accounted for most of the observed effects. As *L. reuteri* is known to enhance the epithelial barrier function of the gut [16, 17], we measured the level of endotoxin in the blood, and found it to be significantly higher in *lpr* mice compared to the age-matched MRL controls (Fig. 2b). Interestingly, increasing colonization of *Lactobacillales* in the gut significantly decreased endotoxemia in *lpr* mice (Fig. 2c). These results suggest that the gut of *lpr* mice may be “leaky” and allow bacterial components (e.g., lipopolysaccharide, or LPS/endotoxin) to enter the blood stream. *L. reuteri* and the uncultured *Lactobacillus* sp., on the other hand, may be able to correct the leakiness. To test if the gut barrier was leaky in *lpr* mice, we gavaged them with FITC-dextran and found significantly more FITC-dextran in the blood compared to MRL mice. When we treated the *lpr* mice with *Lactobacillus* spp., the levels of FITC-dextran in the circulation significantly decreased (Fig. 2d).

Two mucus layers cover the epithelial cells in the lower gastrointestinal tract [18]. Underneath the mucus layers, permeability of the intestinal epithelium is controlled by functions of tight junction proteins [19]. To determine whether *lpr* mice had alterations in epithelial cell junctions, we isolated intestinal epithelial cells and measured the level of tight junction protein transcripts. We found that treatment with *Lactobacillus* spp. significantly increased the expression of barrier-forming junction transcripts (*ZO1*, *occludin*, and *Cldn1*) without affecting the level of pore-forming junction transcript *Cldn2* (Fig. 2e), suggesting enhanced barrier function of the intestinal epithelium with a higher abundance of *Lactobacillales* in the gut microbiota. Immunohistochemical analysis confirmed that the level of ZO-1 was increased by *Lactobacillus* treatment in both the ileum and the colon (Fig. 2f). We also found that epithelial expression of IL-18, a cytokine important for tissue repair [20] and limiting colonic T-helper 17 cell (Th17) differentiation [21], was significantly enhanced with *Lactobacillus* treatment (Fig. 2e). Interestingly, IL-18 can also be detrimental and promote inflammation in *lpr* mice [22]. We found that unlike epithelial expression, the level of IL-18 produced by MLN was significantly decreased by *Lactobacillus* treatment (data not shown). It is likely that *Lactobacillus* spp. can attenuate lupus disease through modulating the production of IL-18 from epithelial vs. immune cells.

In addition to strengthening intestinal mucosal barrier function, *L. reuteri* and the uncultured *Lactobacillus* sp. may also enhance LPS clearance by increasing the expression of intestinal alkaline phosphatase (IAP). IAP is a brush border enzyme expressed on the microvillus membranes of enterocytes [23] that can dephosphorylate LPS, leading to a 100-fold reduction in LPS toxicity [24]. In our studies, the epithelial expression of IAP (*Alppl2* and *Alpi*) was significantly upregulated after *Lactobacillus* treatment in *lpr* mice compared to the controls (Fig. 2g). The upregulation of IAP was confirmed with immunohistochemical analysis (Fig. 2h). Interestingly, IAP has been reported to support the growth of Gram-positive bacteria [25], which may explain the increase of *Bifidobacteria* in *Lactobacillus*-treated mice (Fig. 1a). *Bifidobacteria* can also promote gut epithelial integrity by strengthening tight junctions [26]. Together, these results suggest that gut microbiota can restore intestinal mucosal barrier function that is compromised in lupus-prone *lpr* mice.

### Control of gut inflammation in lupus

With an enhanced gut mucosal barrier, fewer bacteria are able to translocate across the intestinal epithelium leading to reduced activation and migration of CX3CR1^+^ and/or CD103^+^ antigen-presenting cells (APCs) to the draining lymph nodes of the lower intestinal tract [27–29]. The decrease in APC migration may decrease the activation of CD4^±^ T cells. Indeed, we found significantly decreased levels of *Cx3cr1* and *Itgae* (a subunit of CD103) specifically in the MLN with *Lactobacillus* treatment (Additional file 1: Figures S2A and S2B) suggesting that *L. reuteri* and the uncultured *Lactobacillus* sp. may reduce the migration of APC to the MLN. We next determined whether the activation of T cells was affected by the decrease of APC in the MLN. Upon activation, MLN T cells upregulate integrin α4β7 and chemokine receptor CCR9 for homing to the gut mucosa [30]. We found that *Lactobacillus* treatment significantly reduced the expression of both *Itga4* and *Ccr9* in the MLN (Additional file 1: Figures S2B and S2C), suggesting decreased activation of T cells. Consistent with this observation, migration of T cells to the intestinal lamina propria was reduced after mice were treated with the *Lactobacillus* spp. (Additional file 1: Figure S2D).

**Fig. 3:**
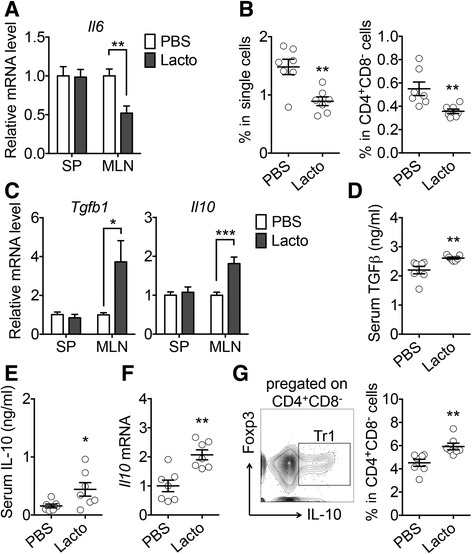
Control of intestinal inflammation by gut microbiota in female *lpr* mice. **a** Transcript level of IL-6 in the spleen (*SP*) and MLN (*n* = 7 mice per group; **P < 0.01). **b** Percentage of IL-6-expressing cells in the MLN (*n* = 7 mice per group; **P < 0.01). **c** Transcript levels of TGFβ and IL-10 (*n* = 7 mice per group; *P < 0.05, ***P < 0.005). **d** Serum level of TGFβ (*n* = 7 mice per group; **P < 0.01). **e** Serum level of IL-10 (*n* = 7 mice per group; *P < 0.05). **f** Transcript level of IL-10 in the kidney (*n* = 7 mice per group; **P < 0.01). **g** FACS analysis of IL-10-expressing Tr1 cells in the MLN. Percentages of Tr1 cells in CD4^+^CD8^−^ cells are shown (*n* = 7 mice per group; **P < 0.01)

Among many pro-inflammatory cytokines produced by activated APC and T cells, IL-6 is known to promote antibody production from B cells [31] and suppress Treg cells [32], which are important for lupus progression in *lpr* mice [33–35]. We measured the transcript level of *Il-6* in the MLN vs. spleen and found that it was significantly reduced by *Lactobacillus* treatment specifically in the MLN (Fig. 3a). CD4^+^CD8^−^ T cells appeared to be a source of IL-6 in the MLN of *lpr* mice (Fig. 3b). As decreased IL-6 would theoretically allow for differentiation of Treg cells [32], we next evaluated the levels of TGFβ and IL-10. Both cytokines were significantly increased at the transcriptional level in the MLN, but not the spleen, with *Lactobacillus* treatment (Fig. 3c), suggesting gut-specific immunosuppression. The serum TGFβ level was also significantly enhanced with the treatment (Fig. 3d), while the level of IL-6 in the circulation did not change (data not shown). Importantly, the induction of IL-10 with more *Lactobacillales* in the gut microbiota was not only in the MLN, but also systemic (Fig. 3e), suggesting that *L. reuteri* and the uncultured *Lactobacillus* sp. may exert a global anti-inflammatory function in *lpr* mice through inducing IL-10 in the gut. Indeed, we also observed a significant elevation of IL-10 transcript levels in the kidney of *lpr* mice with *Lactobacillus* treatment compared to untreated controls (Fig. 3f). Further analysis of MLN cells revealed that most IL-10-producing cells in the gut were CD4^+^Foxp3^−^ type 1 regulatory T (Tr1) cells (Fig. 3g). This observation is consistent with published results on IL-10-producing Tr1 cells in *lpr* mice [36]. Together, these results suggest that gut microbiota can promote an anti-inflammatory environment in the gut of lupus-prone mice, leading to induction of IL-10 that enters the circulation to provide systemic immunosuppression.

### Control of renal inflammation in lupus

**Fig. 4:**
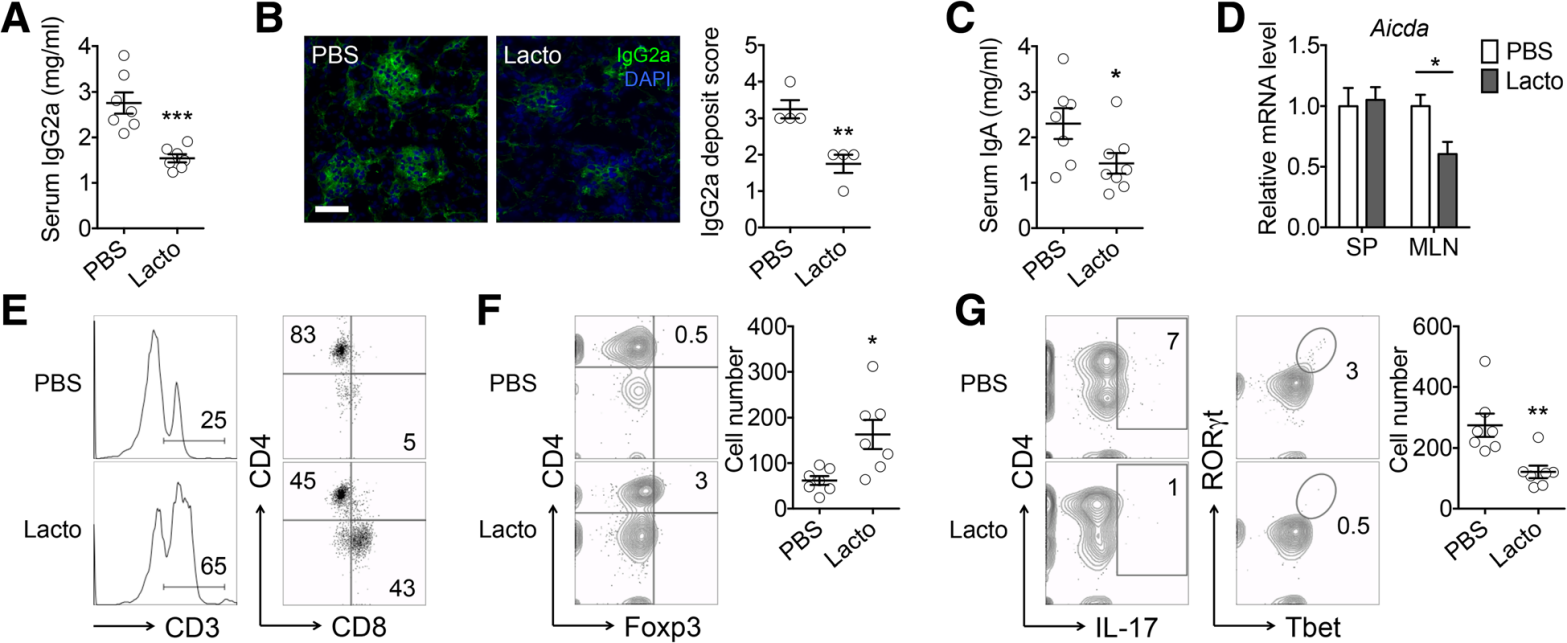
Control of renal inflammation by gut microbiota in female *lpr* mice. **a** Serum level of IgG2a (*n* = 7 mice per group; ***P < 0.005). **b** Immunohistochemical stains of IgG2a (*green*) in the kidney. *Bar* equals 75 μm. Pathological scores are shown on the *right* (*n* = 4 mice per group; **P < 0.01). **c** Serum level of IgA (*n* = 7 or 8 mice per group; *P < 0.05). **d** Transcript level of *Aicda* (*n* = 7 mice per group; *P < 0.05). **e** Percentages of T cells and subpopulations in the kidney. **f** Percentage of CD4^+^Foxp3^+^ Treg cells in the kidney. Absolute Treg cell numbers are shown on the *right* (*n* = 7 mice per group; *P < 0.05). **g** FACS analysis of IL-17-producing CD4^+^ cells and percentage of RORγT^+^Tbet^+^ pathogenic Th17 cells in the kidney. Absolute pathogenic Th17 cell numbers are shown on the *right* (*n* = 7 mice per group; **P < 0.01)

IL-10 can inhibit kidney disease in *lpr* mice through preventing IFNγ-mediated production of IgG2a, a major immune deposit in the kidney of these mice [37]. We found that *Lactobacillus* treatment significantly reduced the level of IgG2a in the blood (Fig. 4a) and its deposition in the kidney (Fig. 4b). This suggests that IgG2a may act as another “messenger” (in addition to IL-10) to transduce the disease-modulating signal from the gut to the kidney. The levels of IgG1 and total IgG did not change with the treatment (data not shown). Interestingly, the level of IgA was reduced by *Lactobacillus* treatment in the circulation (Fig. 4c), suggesting a potential effect of *L. reuteri* and the uncultured *Lactobacillus* sp. on class-switched antibodies. Indeed, the expression level of *Aicda*, whose gene product mediates class switch recombination [38], was significantly lower in the MLN of *lpr* mice treated with *Lactobacillus* spp. (Fig. 4d). The change of IgA did not appear to be related to attenuation of LN, as it was not detectable in the kidney.

Different immune cell populations, including T, B, neutrophils, dendritic cells, and macrophages, have been demonstrated to infiltrate in the kidney with LN. To determine how *Lactobacillus* treatment affects immune cell migration to renal tissue, we evaluated various immune cell populations and found marked influx of CD3^+^ T cells, particularly CD8^+^ T cells, into the kidney of *Lactobacillus*-treated *lpr* mice (Fig. 4e). As CD8^+^ T cells are generally considered protective in lupus [39–41], it would suggest that renal infiltration of these cells exerts a suppressive effect on the development of LN. In addition, the number of Foxp3^+^ Treg cells significantly increased (Fig. 4f), while that of pathogenic Th17 cells significantly decreased (Fig. 4g), with *Lactobacillus* treatment. Together, these results suggest that gut microbiota may attenuate LN by limiting renal deposition of IgG2a and skewing the Treg-Th17 balance in the kidney towards Treg.

### Sex hormones and gut microbiota cooperatively regulate LN

**Fig. 5:**
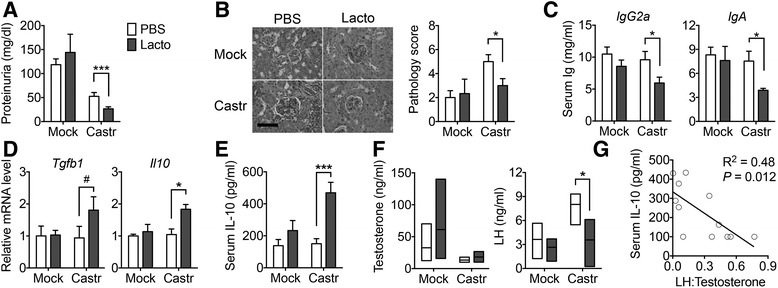
Sex hormones and gut microbiota cooperatively regulate LN. **a** Proteinuria after surgery (*Mock* vs. *Castr*/castration) and treatment (*PBS* vs. *Lacto*) of male *lpr* mice (*n* = 5 mice per group; Mann-Whitney test; ***P < 0.005). **b** Renal histopathology (*n* = 5 mice per group; Mann-Whitney test; *P < 0.05). *Left*: PAS-stained kidney sections; *bar* equals 100 μm. **c** Serum levels of IgG2a and IgA (*n* = 5 mice per group; *P < 0.05). **d** Transcript levels of TGFβ and IL-10 in the MLN (*n* = 5 mice per group; ^#^P < 0.1, *P < 0.05). **e** Serum level of IL-10 (*n* = 5 mice per group; ***P < 0.005). **f** Levels of testosterone and luteinizing hormone (*LH*) in the blood (*n* = 3 mice per group; *P < 0.05). **g** Negative correlation between serum IL-10 and the ratio of LH to testosterone

SLE is a female-biased disease with women getting disease nearly 9:1 over men. The results shown so far were obtained from female mice. However, in lpr mice, both sexes get LN similarly. To investigate whether sex hormones and gut microbiota cooperatively regulate LN in *lpr* mice, we treated male mice with the same *Lactobacillus* strains after mock or castration surgery (Additional file 1: Figure S3A). Bacterial profiling showed that *Lactobacillus* treatment increased the gut colonization of *Lactobacillales* in both mock and castrated mice (Additional file 1: Figure S3B and Additional file 3: Table S2). Strikingly, *Lactobacillus* treatment significantly decreased proteinuria (Fig. 5a) and renal pathology (Fig. 5b) only in the castrated mice but not the intact animals, suggesting a possible role of androgenic hormones in suppressing the effects of *Lactobacillus* spp. The level of anti-double-stranded DNA (anti-dsDNA) IgG was not changed with *Lactobacillus* treatment (Additional file 1: Figure S3C). However, the total weight of lymph nodes (including mesenteric, renal, inguinal, lumbar, superficial, axillary/brachial, mediastinal lymph nodes) increased after mice were castrated, an effect reversed by *Lactobacillus* treatment (Additional file 1: Figure S3D). In addition, increasing gut colonization of *Lactobacillales* significantly decreased the serum levels of IgG2a and IgA in castrated male mice, but not in the mice receiving mock surgery (Fig. 5c). The decrease of IgA appears to have originated from the colon (Additional file 1: Figure S3E), where the majority of *Lactobacillus* spp. (in terms of total number) resided [42]. Importantly, we found that unlike mice receiving in mock surgery, *Lactobacillus* treatment significantly increased the transcript levels of TGFβ and IL-10 in the MLN in castrated male *lpr* mice (Fig. 5d). *Lactobacillus* treatment also significantly increased circulating IL-10 in castrated animals only (Fig. 5e). Together, these results suggest that *Lactobacillus* treatment was not effective in intact male *lpr* mice, while the response of castrated males to *Lactobacillus* treatment parallels that of female *lpr* mice.

As testis is the only source of testosterone in mice, castration surgery completely removed the male hormone regardless of *Lactobacillus* treatment (Fig. 5f). We then measured two hormones regulated by testosterone, luteinizing hormone (LH), and follicle-stimulating hormone (FSH). Both are known to be repressed by testosterone [43–45]. As anticipated, castration surgery increased the levels of LH and FSH when the mice were not treated with *Lactobacilli* (Fig. 5f and Additional file 1: Figure S3F). However, *Lactobacillus* treatment significantly decreased the serum level of LH, bringing it back to the level where testosterone was still present. We took the ratio of LH to testosterone and found it to be negatively correlated with serum IL-10 level (Fig. 5g). Whether LH directly affects IL-10, or vice versa, requires further investigation. Together, these results suggest that gut microbiota control LN in *lpr* mice in a sex hormone-dependent manner. To determine the effect of *Lactobacilli* on sex differences, in future studies, we will transfer the cecal contents of young females to male mice to determine whether the interaction between sex hormones and *Lactobacillus* treatment is required for the observed changes in autoimmune response and/or disease phenotype.

## Discussion

The goal of this study was to understand the role of gut microbiota in the pathogenesis of SLE-associated kidney inflammation. In the *lpr* model of LN, we found marked depletion of *Lactobacillales* in the gut microbiota compared to MRL controls. Increasing *Lactobacillales* in the gut microbiota improved the renal function of *lpr* mice. Since *Lactobacillus* spp. are known to enhance the mucosal barrier function, the level of circulating endotoxin was measured. Endotoxin can accelerate nephritis in lupus-prone mice [46–48], and significantly higher endotoxemia was observed in *lpr* mice preceding the onset of kidney disease. This suggests a “leaky gut” in pre-disease *lpr* mice. *Lactobacillus* treatment significantly decreased intestinal permeability in these mice and likely prevented detrimental bacteria and their antigens from penetrating the intestinal epithelium. *Lactobacillus* treatment also decreased CX3CR1 and CD103 expression in the MLN. CX3CR1- and CD103-expressing cells are primarily APC [29, 49, 50] that can capture bacteria from the gut lumen and transport them to the MLN, where they present antigens and activate CD4^+^ T cells to produce IL-6 that suppresses Treg, which is vital to lupus pathogenesis in *lpr* mice. By preventing barrier compromise and decreasing microbial translocation, increased gut colonization of *Lactobacillus* spp. may reduce activation and migration of APC to the MLN, hence suppressing IL-6 production and allowing for Foxp3^−^ Tr1 cells to produce IL-10, which subsequently represses the synthesis and renal deposition of IgG2a. Inside the kidney, the Treg-Th17 balance was skewed towards Treg with *Lactobacillus* treatment. These effects of *Lactobacilli*, illustrated in Additional file 1: Figure S4, were absent in male mice unless castrated, suggesting that gut microbiota attenuates LN in a sex hormone-dependent manner. It is noteworthy that in these experiments, *Lactobacillus* treatment was given before disease establishment. It appears that *L. reuteri* and the uncultured *Lactobacillus* sp. have a preventative instead of curative effect on the development of LN.

Compromised intestinal barrier function has been reported in autoimmune conditions such as the inflammatory bowel disease (IBD), which includes Crohn’s disease (CD) and ulcerative colitis (UC). It has been shown by using cecal biopsies that intestinal permeability is significantly increased in both CD and UC patients with irritable bowel syndrome-like symptoms than those with quiescent IBD without the symptoms [51]. This increase was accompanied by downregulation of the tight junction protein ZO-1. Endotoxemia in SLE patients that suggests disrupted gut mucosal barrier function in human SLE has also been reported [52]. In our studies, we show that the intestinal epithelium is compromised in lupus-prone *lpr* mice and that *Lactobacillus* treatment can restore mucosal barrier function by increasing the expression of ZO-1. The effect of *Lactobacilli* on gut barrier function may also be attributed to the increase of Muc2, a mucin protein secreted by goblet cells that functions primarily to protect the intestinal epithelium [53].

The imbalance between anti-inflammatory Treg and inflammatory Th17 cells is widely recognized as being causative in the onset of both murine lupus and human SLE [54]. It is well established that environmental factors can promote plasticity between Treg and Th17 cells including the presence of inflammatory cytokines [55]. This cellular flexibility is due to effects of these inflammatory cytokines on the expression and function of the lineage-defining transcription factors Foxp3 and RORγt, which promote Treg and Th17 cell fates, respectively [56, 57]. Intriguingly, changes in the composition of gut microbiota, particularly those of *Clostridia* and segmented filamentous bacteria (SFB) in mice and *Bacteroides fragilis* in humans, have been shown to alter the balance between Treg and Th17 cells [12]. We show here that increasing *Lactobacillales* in the gut microbiota can promote renal Treg cells and suppress disease-causing Th17 cells to attenuate kidney inflammation in lupus-prone mice.

## Conclusions

Environmental triggers initiate SLE in susceptible individuals. Since the gastrointestinal system serves as a first line of defense against various pathogens, delineating the type of flora and understanding the role the microbiota plays in determining disease susceptibility in SLE patients are paramount. We show in lupus-prone mice that *Lactobacillus* spp. in the gut microbiota exert anti-inflammatory effects by repairing the damaged gut barrier, suppressing pro-inflammatory factors in the lymphatic circulation, and improving the ratio of regulatory versus pathogenic T cells, thereby attenuating kidney inflammation. While the relative abundance of *Lactobacillales* appears to be normal in SLE patients in remission (without active disease) [5], this does not preclude the possibility that beneficial bacteria capable of strengthening the gut barrier are lacking in SLE patients with active disease, especially those with LN. SLE is a very diverse disease; therefore, it is important to separately analyze the gut microbiota of SLE patients with different clinical manifestations. If the results of our mouse studies that *L. reuteri* and the uncultured *Lactobacillus* sp. which have a preventative effect on the development of LN can be replicated in humans, this may be a new avenue to identify at-risk individuals and provide protection in SLE-prone populations.

## Methods

### Mice

MRL/Mp (MRL), MRL/Mp-*Fas* ^*lpr*^ (MRL/*lpr* or *lpr*, stock number 000485) mice were purchased from The Jackson Laboratory (Bar Harbor, ME) and bred and maintained in a specific-pathogen-free facility according to the requirements of the Institutional Animal Care and Use Committee at Virginia Polytechnic Institute and State university. Reciprocal cecal microbiota transplantation experiments were performed by diluting, under anaerobic conditions, contents of a cecum collected from one 3-week-old MRL or MRL/lpr donor mouse in 5 mL PBS. The cecal material was then suspended by vortexing, and the suspension was introduced by oral gavage into recipient mice at 0.2 mL/mouse when the mice were 3 weeks old and weaned. Another donor mouse was sacrificed on the next day, and the same procedure was repeated once. All *Lactobacillus* strains, including *L. oris* (F0423), *L. rhamnosus* (LMS201), *L. reuteri* (CF48-3A), *L. johnsonii* (135-1-CHN), and *L. gasseri* (JV-V03), were obtained from BEI Resources. All 5 strains were freshly cultured every week, mixed, and inoculated to MRL/lpr mice from 3 weeks old of age until dissection. For the experiment involving male castration, the testes and epididymis were removed through a scrotal incision under isoflurane inhalant anesthesia. The skin was closed using wound clips. Mock orchidectomy was performed on an equal number of mice to serve as surgical controls. The mock group of mice were prepared and anesthetized, and a scrotal incision was made; however, the incision was closed with a wound clip, without gonad removal. All mice were administered ketoprofen, diluted to 0.5 mg/mL in sterile PBS, subcutaneously at 3.5 mg/kg as an analgesic post-operatively.

### Microbiota sampling and analysis

Fecal microbiota samples were obtained by taking individual mice out of their cage and collecting a fecal pellet. To avoid cross-contamination, each microbiota sample was collected by using a new pair of sterile tweezers. Samples were stored at −80 °C till being processed at the same time. Sample homogenization, cell lysis, and DNA extraction were performed as previously described [13]. PCR were performed, and purified amplicons were sequenced bidirectionally on an Illumina MiSeq at Argonne National Laboratory.

### Renal function

Urine was collected biweekly, and all samples were stored at −20 °C till being analyzed at the same time with a Pierce Coomassie Protein Assay Kit (Thermo Scientific). When the mice were euthanized at 14 weeks of age, the kidneys were fixed in formalin for 24 h, paraffin embedded, sectioned, and stained with periodic acid-Schiff (PAS) at the Histopathology Laboratory at Virginia Maryland Regional College of Veterinary Medicine. Slides were read with an Olympus BX43 microscope. All the slides were scored in a blinded fashion by a certified veterinary pathologist. Glomerular lesions were graded on a scale of 0 to 3 for each of the following 5 categories: increased cellularity, increased mesangial matrix, necrosis, the percentage of sclerotic glomeruli, and the presence of crescents. Tubulointerstitial lesions were graded on a scale of 0 to 3 for each of the following four categories: presence of peritubular mononuclear infiltrates, tubular damage, interstitial fibrosis, and vasculitis.

### Endotoxin quantification and ELISA

Separated serum after blood clotting was saved at −20 °C until use. Serum endotoxin level was measured by using a Pierce LAL Chromogenic Endotoxin Quantitation Kit (Thermo Scientific). Anti-dsDNA IgG was measured according to a previously described method [58]. Serum IgG, IgA, IgG2a, and IL-10 concentrations were determined with mouse IgG, IgA, IgG2a (Bethyl Laboratories), and IL-10 (Biolegend) ELISA kits, respectively, according to the manufacturers’ instructions.

### Immunohistochemistry

The kidneys and 0.5-cm-length ileal and colonic sections were embedded in Tissue-Tek O.C.T. Compound (Sakura Finetek) and rapidly frozen in a freezing bath of dry ice and 2-methylbutane. Frozen OCT samples were cryosectioned and unstained slides were stored at −80 °C. Frozen slides were warmed to room temperature and let dry for 30 min, followed by fixation in −20 °C cold acetone at room temperature for 10 min. After washing in PBS, the slides were blocked with PBS containing 1% BSA for 20 min at room temperature. The slides were then incubated with fluorochrome-conjugated antibody mixture at room temperature in a dark humid box. The slides were mounted with Prolong Gold containing DAPI (Life Technologies). The following antibodies were used in immunohistochemical analysis: anti-mouse IgG2a-FITC (eBiosciense), rabbit anti-mouse ZO-1 and FITC-conjugated goat anti-rabbit IgG secondary antibody (Thermo Scientific), and rabbit anti-mouse IAP primary antibody (GeneTex). The slides were read and pictured with EVOS FL microscope (Advanced Microscopy Group) and a ×20 objective.

### Intestinal permeability

In vivo intestinal permeability assay was performed by using FITC-conjugated dextran (Sigma-Aldrich). Briefly, mice were deprived of water overnight and then orally gavaged with FITC-dextran dissolved in PBS at 40 mg/100 g body weight (around 300 μL/mouse). Mice were anesthetized after 4 h, and the blood was collected and saved in the dark until serum separation. Serum was then diluted 1:1 with PBS and added to a 96-well microplate in duplicate, followed by determination of FITC concentration with Glomax (Promega) at an excitation of 485 nm and an emission wavelength of 528 nm using serially diluted FITC-dextran as the standard.

### Organ cultures

Ileum and colon of 1 cm length were collected and opened longitudinally. Intestinal sections were thoroughly washed by PBS and cultured in 48-well plate with 500 μL C10 media at 37 °C. Supernatant was collected after 24 h and analyzed by using ELISA.

### RT-quantitative PCR

The spleen, MLN, and isolated intestinal epithelial cells (IECs; see below for isolation procedure) were homogenized with Bullet Blender homogenizer (Next Advance), and total RNA was extracted with RNeasy Plus Mini Kit (Qiagen) according to the manufacturers’ instructions. Genomic DNA was removed by digestion with RNase-free DNase I (Qiagen). Reverse transcription was performed by using iScript cDNA Synthesis Kit (Bio-Rad). Quantitative PCR was performed with iTaq Universal SYBR Green Supermix (Bio-Rad) and ABI 7500 Fast Real-Time PCR System (Applied Biosystems). Relative quantities were calculated using *L32* (MLN and spleen) and *Villin* (IECs) as the housekeeping gene. Primer sequences for mouse *L32*, *Villin*, *ZO1*, *Occludin*, *Cldn1*, *Cldn2*, *IL18*, *IL6*, *Tgfb1*, *IL10*, *Acida*, *CX3CR1*, *CCR9*, *Itgae*, *Itgb7*, and *Itga4* are available upon request.

### Cell isolation and flow cytometry

The spleen, MLN, and Peyer’s patches were collected and mashed in 70-μm cell strainers with C10 media. For splenocytes, red blood cells were lysed with RBC lysis buffer (eBioscience). To isolate lamina propria lymphocytes, the intestine was opened longitudinally and cut into pieces. The pieces were incubated twice in EDTA-DTT solution and intensively vortexed to remove the epithelial cell layer (saved as IEC-enriched fractions). After the second EDTA incubation, the pieces were cut and placed in a digestion solution containing 1 mg/mL collagenase D (Roche), 0.1 mg/mL DNase I (Sigma), and 10 μg/mL Dispase (Fisher). After digestion, the solution was passed through a 100-μm cell strainer. The same process was repeated three times, and the supernatants of the three digestions were combined and added onto a 40:80 Percoll gradient to separate lymphocytes [59]. For surface marker staining, the cells were blocked by anti-mouse CD16/32 (eBioscience), stained with fluorochrome-conjugated antibodies, and analyzed with Attune NxT flow cytometer (Thermo Scientific). For intracellular staining, Foxp3 Fixation/Permeabilization kit (eBioscience) was used. Anti-mouse antibodies used in this study include the following: CD3-APC-eFluor 780, IL-6-FITC, CD8-PE-Cy7, Tbet-PerCP-Cy5.5, CD4-PerCP-Cy5.5, and RORγT-PE (eBioscience); CD45-FITC, Foxp3-Alexa Fluor 647, IL-10-BV421, and IL-17A-APC (Biolegend); and CD19-PerCP-Cy5.5, CD4-PE-Cy7, and CD8a-V450 (BD Biosciences). Flow cytometry data were analyzed with FlowJo.

### Hormone measurements

Serum samples were saved at −80 °C until analysis. Testosterone, luteinizing hormone, and follicle-stimulating hormone were measured at the University of Virginia Center for Research in Reproduction, which is supported by the Eunice Kennedy Shriver NICHD/NIH (NCTRI) Grant P50-HD28934.

### Statistical analysis

For the comparison of two groups, unpaired Student’s *t* test was used unless specified. For the comparison of more than two groups, one-way ANOVA and Tukey’s post-test were used. Results were considered statistically significant when *P* < 0.05 (*P < 0.05, **P < 0.01, ***P < 0.005). All analyses were performed with Prism GraphPad.

## Additional files

Additional file 1: Figure S1-S4. (A) Relative abundance of Lactobacillaceae in fecal microbiota (*n* = 4 per group; *P < 0.05 at 5 weeks of age). (B) Study design of cecal transplantation from MRL to lpr mice. (C) Level of anti-dsDNA IgG produced by 1-cm-long ileal or colonic organ culture after 24-h incubation (*n* > 3 per group; *P < 0.05). (D) Time-dependent changes of fecal microbiota upon cecal transplantation. Abundant bacterial OTU (>0.1%) were summarized (*n* = 4 per group). (E) Study design of *Lactobacillus* treatment of lpr mice. (F–G) Female MRL/lpr mice were treated with PBS control or mixed antibiotics (Abx) for 2 days at 3 weeks of age and sacrificed at 14 weeks of age (*n* = 3 per group). The levels of proteinuria (F) and anti-dsDNA antibodies (G) at 14 weeks of age are shown. The differences were not significant. (H) Weight of spleen and MLN of lpr mice upon *Lactobacillus* treatment. Figure S2. (A) Transcript level of CX3CR1 in lymphoid tissues of lpr mice treated with PBS or *Lactobacilli*. (B) Transcript levels of CD103 (Itgae and Itgb7) and a4b7 (Itga4 and Itgb7). (C) Transcript level of CCR9. (D) Percentage of CD3+ T cells in the intestinal lamina propria. *P < 0.05, **P < 0.01, ***P < 0.001. Figure S3. (A) Study design of surgery and treatment in male lpr mice. (B) Time-dependent changes of fecal microbiota. *Castr* castration. (C) Level of anti-dsDNA IgG in the blood (*n* = 5 per group). (D) Total weight of lymph nodes (LN) from multiple sites (**P < 0.01). (E) Level of IgA produced by 1-cm sections of ileal or colonic organ culture after 24-h incubation (*P < 0.05). (F) Level of FSH in the blood. Figure S4. Working model (see text for details). (PDF 794 kb)

Additional file 2: Table S1. Actual abundance number and *P* values for Fig. 1a. (PDF 30 kb)

Additional file 3: Table S2. Actual abundance numbers and *P* values for Additional file 1: Figure S3B. (PDF 40 kb)

## Abbreviations

CD: Crohn’s disease
dsDNA: Double-stranded DNA
IAP: Intestinal alkaline phosphatase
IBD: Inflammatory bowel disease
IECs: Intestinal epithelial cells
LN: Lupus nephritis
*lpr*: MRL/*lpr*
LPS: Lipopolysaccharide
MLN: Mesenteric lymph node
PAS: Periodic acid-Schiff
SFB: Segmented filamentous bacteria
SLE: Systemic lupus erythematosus
Th17: T-helper 17 cells
Tr1: Type 1 regulatory T cells
Treg: Regulatory T cells
UC: Ulcerative colitis
