# Supplementary material for: Control of lupus nephritis by changes of gut microbiota
Source: Microbiome. 2017 Jul 11;5:73. doi: 10.1186/s40168-017-0300-8 (PMC5505136; doi:10.1186/s40168-017-0300-8)
Supplement: Supplementary file 1 — (A) Relative abundance of Lactobacillaceae in fecal microbiota (n = 4 per group; *P < 0.05 at 5 weeks of age). (B) Study design of cecal transplantation from MRL to lpr mice. (C) Level of anti-dsDNA IgG produced by 1-cm-long ileal or colonic organ culture after 24-h incubation (n > 3 per group; *P < 0.05). (D) Time-dependent changes of fecal microbiota upon cecal transplantation. Abundant bacterial OTU (>0.1%) were summarized (n = 4 per group). (E) Study design of Lactobacillus treatment of lpr mice. (F–G) Female MRL/lpr mice were treated with PBS control or mixed antibiotics (Abx) for 2 days at 3 weeks of age and sacrificed at 14 weeks of age (n = 3 per group). The levels of proteinuria (F) and anti-dsDNA antibodies (G) at 14 weeks of age are shown. The differences were not significant. (H) Weight of spleen and MLN of lpr mice upon Lactobacillus treatment. Figure S2. (A) Transcript level of CX3CR1 in lymphoid tissues of lpr mice treated with PBS or Lactobacilli. (B) Transcript levels of CD103 (Itgae and Itgb7) and a4b7 (Itga4 and Itgb7). (C) Transcript level of CCR9. (D) Percentage of CD3+ T cells in the intestinal lamina propria. *P < 0.05, **P < 0.01, ***P < 0.001. Figure S3. (A) Study design of surgery and treatment in male lpr mice. (B) Time-dependent changes of fecal microbiota. Castr castration. (C) Level of anti-dsDNA IgG in the blood (n = 5 per group). (D) Total weight of lymph nodes (LN) from multiple sites (**P < 0.01). (E) Level of IgA produced by 1-cm sections of ileal or colonic organ culture after 24-h incubation (*P < 0.05). (F) Level of FSH in the blood. Figure S4. Working model (see text for details). (PDF 794 kb) [file 40168_2017_300_MOESM1_ESM.pdf]

Supplemental Data

Control of lupus nephritis by the gut microbiota

(Mu et al.)

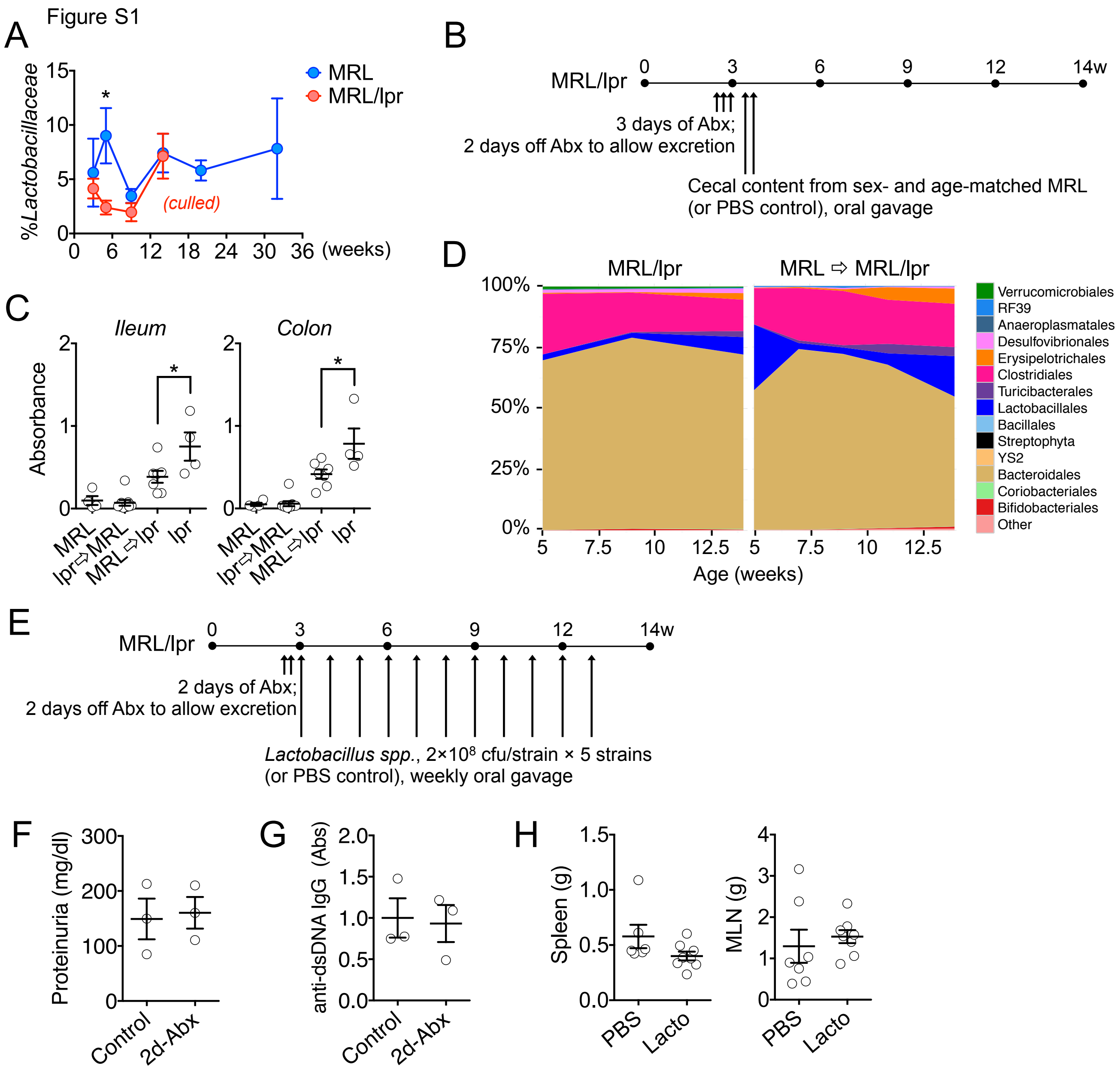

Figure S1. (**A**) Relative abundance of *Lactobacillaceae* in fecal microbiota (n=4 per group; *P<0.05 at 5 weeks of age). (**B**) Study design of cecal transplantation from MRL to *lpr* mice. (**C**) Level of anti-dsDNA IgG produced by 1-cm long ileal or colonic organ culture after 24 h incubation (n>3 per group; *P<0.05). (**D**) Time-dependent changes of fecal microbiota upon cecal transplantation. Abundant bacterial OTU (>0.1%) were summarized (n=4 per group). (**E**) Study design of *Lactobacillus* treatment of *lpr* mice. (**F-G**) Female MRL/lpr mice were treated with PBS control or mixed antibiotics (Abx) for 2 days at 3 weeks of age and sacrificed at 14 weeks of age (n=3 per group). The levels of proteinuria (F) and anti-dsDNA antibodies (G) at 14 weeks of age are shown. The differences were not significant. (**H**) Weight of spleen and MLN of *lpr* mice upon *Lactobacillus* treatment.

Figure S2

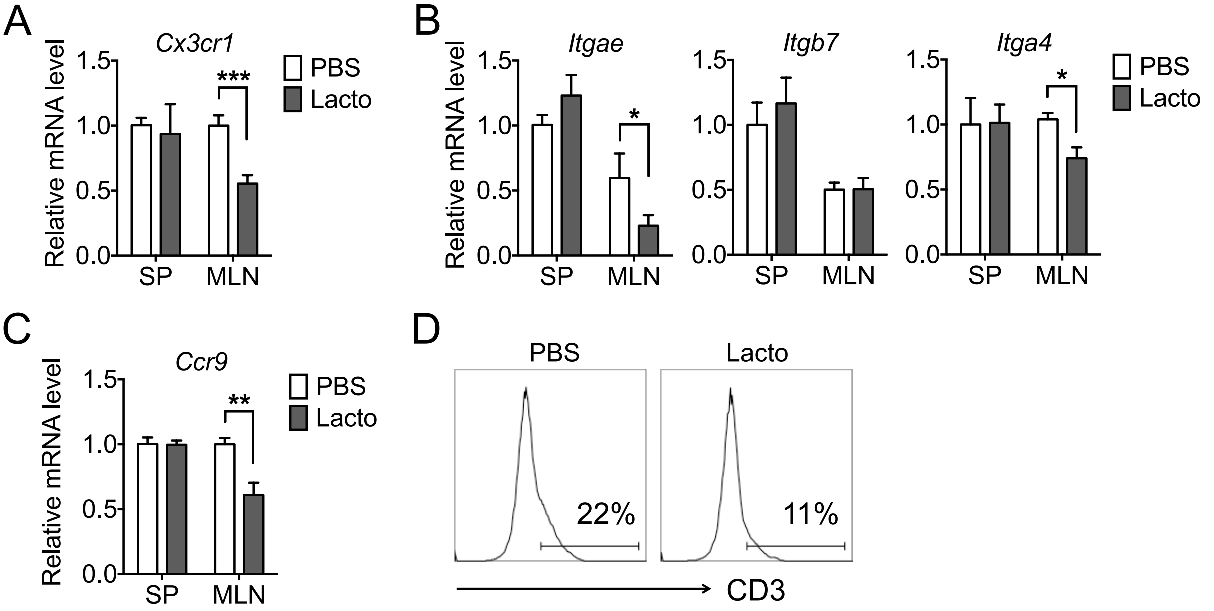

Figure S2. (**A**) Transcript level of CX3CR1 in lymphoid tissues of *lpr* mice treated with PBS or *Lactobacilli*. (**B**) Transcript levels of CD103 (*Itgae* and *Itgb7*) and α4β7 (*Itga4* and *Itgb7*). (**C**) Transcript level of CCR9. (**D**) Percentage of $CD3^+$ T cells in the intestinal lamina propria. *P<0.05, **P<0.01, ***P<0.001.

Figure S3

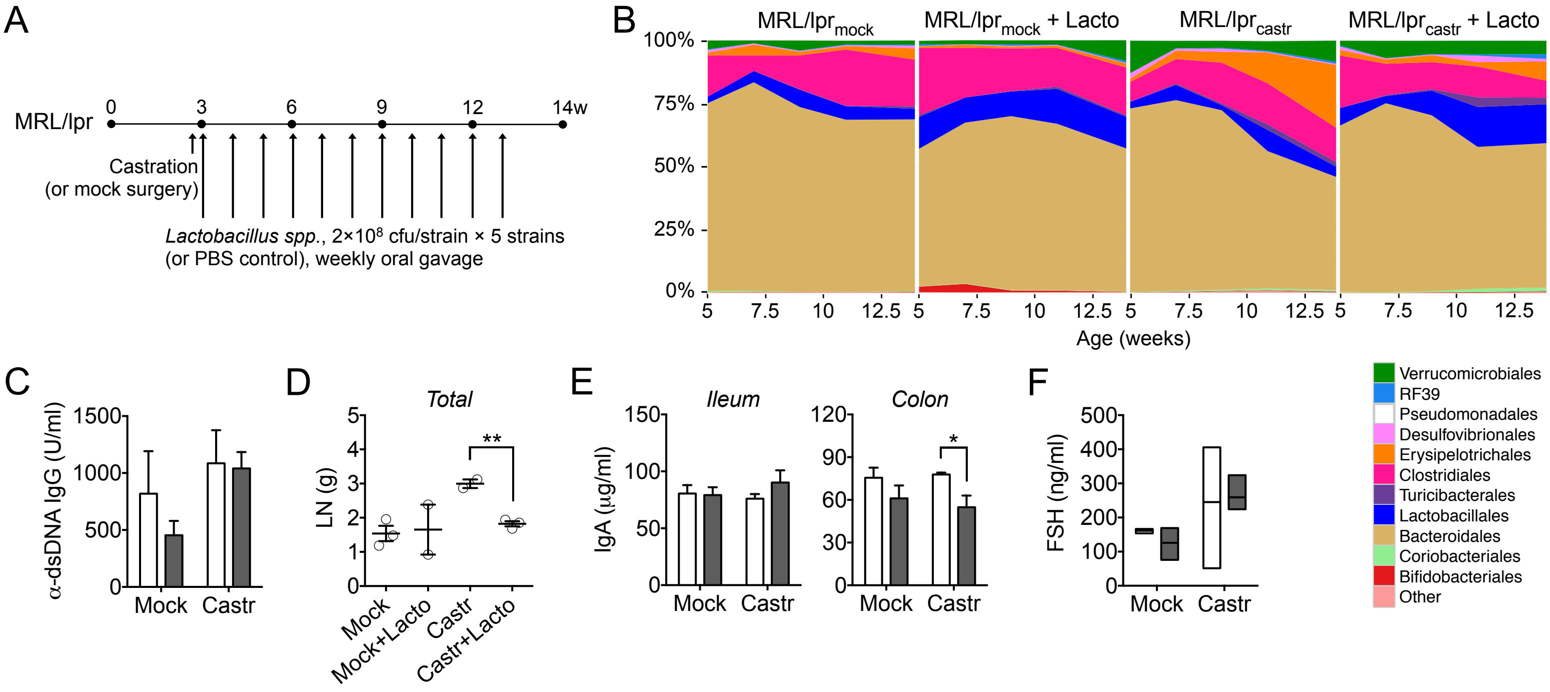

Figure S3. (**A**) Study design of surgery and treatment in male *lpr* mice. (**B**) Time-dependent changes of fecal microbiota. Castr, castration. (**C**) Level of anti-dsDNA IgG in the blood (n=5 per group). (**D**) Total weight of lymph nodes (LN) from multiple sites (**P<0.01). (**E**) Level of IgA produced by 1-cm sections of ileal or colonic organ culture after 24 h incubation (*P<0.05). (**F**) Level of FSH in the blood.

Figure S4

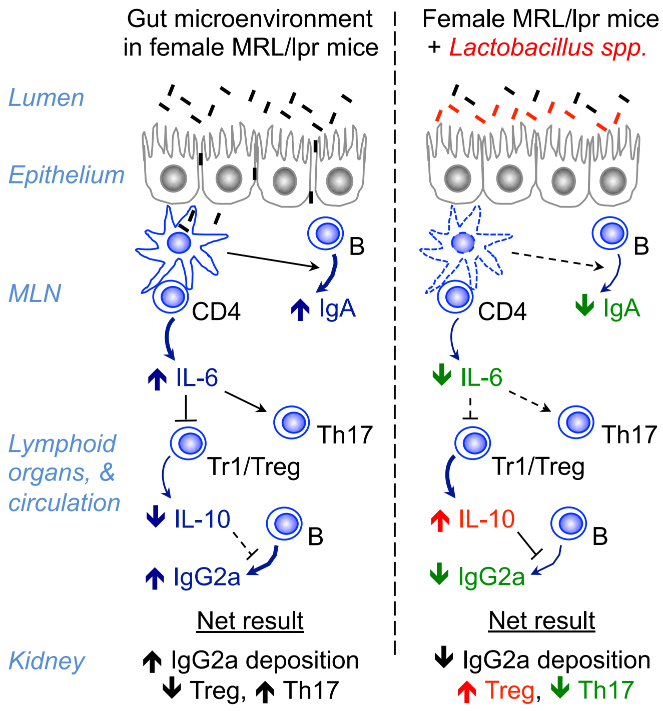

Figure S4. Working model (see text for details).
