# Supplementary material for: Control of lupus nephritis by changes of gut microbiota
Source: Microbiome. 2017 Jul 11;5:73. doi: 10.1186/s40168-017-0300-8 (PMC5505136; doi:10.1186/s40168-017-0300-8)
Supplement: Supplementary file 2 — Actual abundance number and P values for Fig. 1a. (PDF 30 kb) [file 40168_2017_300_MOESM2_ESM.pdf]

Table S1. Actual abundance number and P values for Figure 1A.

Weeks	5				
**Treatment**	**PBS**		**Lacto**		
**Statistics**	**MEAN**	**SEM**	**MEAN**	**SEM**	**p value**
Other	0.001452573	0.000370512	0.00216467	0.000611468	0.384227986
Bifidobacteriales	0.000092862	3.92E-05	0.02107776	7.61E-03	0.110273902
Coriobacteriales	0.000619615	0.000367978	0.000702969	0.000155201	0.84493291
Bacteroidales	0.5401281	0.123787605	0.47425	0.020228301	0.634076753
Streptophyta	0.00E+00	0.00E+00	3.85E-04	1.50E-04	0.124598367
Bacillales	0.00E+00	0.00E+00	4.36E-05	4.36E-05	0.422649731
Lactobacillales	0.03588617	0.012585734	0.18035717	0.032055487	0.032036483
Turicibacterales	0.00011632	2.41E-05	0.006149833	3.17E-03	0.19775303
Clostridiales	0.40060196	0.125280413	0.2721557	0.017436391	0.382132001
Erysipelotrichales	0.003531374	0.002670929	0.002559914	0.000878528	0.74884078
Desulfovibrionales	0.012642986	0.008916869	0.0000218	0.0000218	0.251894434
Anaeroplasmatales	7.36E-05	7.36E-05	1.88E-02	1.80E-02	0.406822606
RF39	0.001854032	1.85E-03	0.010568362	5.06E-03	0.220497569
Verrucomicrobiales	0.001006175	0.000445068	0.009579891	0.004264091	0.180799451

Weeks	7				
**Treatment**	**PBS**		**Lacto**		
**Statistics**	**MEAN**	**SEM**	**MEAN**	**SEM**	**p value**
Other	0.000524403	0.000150731	0.002737741	0.000726588	0.087234024
Bifidobacteriales	0.000627973	1.50E-04	0.011039188	3.65E-03	0.103643606
Coriobacteriales	0.001299495	0.000184289	0.00095909	0.000311355	0.409186181
Bacteroidales	0.8885538	0.009011444	0.48228	0.056124398	0.01657491
Streptophyta	0.00E+00	0.00E+00	3.06E-04	2.27E-04	0.309581077
Bacillales	0.00E+00	0.00E+00	1.35E-04	1.39E-05	0.010427194
Lactobacillales	0.0172074	0.002096525	0.12035304	0.013731074	0.015537088
Turicibacterales	0.000109734	8.86E-05	0.037399986	9.17E-03	0.0554803
Clostridiales	0.03873227	0.006289114	0.28855133	0.042499258	0.025623019
Erysipelotrichales	0.006706814	0.003302802	0.000984563	0.000159452	0.181545385
Desulfovibrionales	0.004205423	0.000753264	0	0	0.011346626
Anaeroplasmatales	0.00E+00	0.00E+00	1.71E-03	5.89E-04	0.101267906
RF39	0.000183998	8.40E-05	0.01432384	2.64E-03	0.032954713
Verrucomicrobiales	0.041564902	0.013463065	0.037422723	0.004943481	0.787935912

Weeks	9				
**Treatment**	**PBS**		**Lacto**		
**Statistics**	**MEAN**	**SEM**	**MEAN**	**SEM**	**p value**
Other	0.001179841	0.000215487	0.002060316	0.00060893	0.28237675
Bifidobacteriales	0.000173265	3.64E-05	0.020236536	1.35E-02	0.275678213
Coriobacteriales	0.000572581	0.000167994	0.00087324	0.000603636	0.672949478
Bacteroidales	0.7684187	0.067381031	0.7274715	0.143177924	0.813148092
Streptophyta	3.31E-05	3.31E-05	3.30E-05	3.30E-05	0.99902676
Bacillales	0.00E+00	0.00E+00	1.84E-05	1.84E-05	0.422649731
Lactobacillales	0.01445834	0.004780232	0.04774062	0.024813723	0.310678136
Turicibacterales	0	0.00E+00	0.012634576	6.84E-03	0.206118694
Clostridiales	0.19102699	0.065791882	0.09272055	0.067204723	0.346068818
Erysipelotrichales	0.002734678	0.000913612	0.063304566	0.025064664	0.13674604
Desulfovibrionales	0.009829324	0.002530869	0.002063896	0.002036358	0.062356017
Anaeroplasmatales	1.65E-05	1.65E-05	5.52E-05	5.52E-05	0.561822261
RF39	0.000713047	1.68E-04	0.004125837	2.41E-03	0.292029554

Verrucomicrobiales	0.010012565	0.004005088	0.026427306	0.012246911	0.310820363

**Weeks**	**11**				
**Treatment**	**PBS**		**Lacto**		
**Statistics**	**MEAN**	**SEM**	**MEAN**	**SEM**	**p value**
Other	0.0015511	0.000422145	0.005397532	0.001573434	0.126031396
Bifidobacteriales	0.000963766	2.35E-04	0.030241667	5.72E-03	0.035964901
Coriobacteriales	0.000948233	0.000124655	0.000752113	0.000173865	0.412418221
Bacteroidales	0.8577791	0.04436129	0.5344842	0.060398192	0.012714167
Streptophyta	0.00E+00	0.00E+00	6.88E-05	6.88E-05	0.422649731
Bacillales	0.00E+00	0.00E+00	0.00E+00	0.00E+00	NA
Lactobacillales	0.04488269	0.006875729	0.08236474	0.017565435	0.154292602
Turicibacterales	0.000014425	1.44E-05	0.017864688	7.61E-03	0.143545561
Clostridiales	0.05749012	0.025185905	0.16563324	0.018845935	0.018684562
Erysipelotrichales	0.001799617	0.00065857	0.06854392	0.030589244	0.160784098
Desulfovibrionales	0.008013002	0.001295393	0	0	0.008508772
Anaeroplasmatales	2.77E-05	2.77E-05	3.41E-03	1.29E-03	0.119946046
RF39	0.0006894	3.40E-04	0.006711107	3.07E-03	0.187114942
Verrucomicrobiales	0.025698467	0.014244709	0.082181708	0.012088862	0.029361225

**Weeks**	**14**				
**Treatment**	**PBS**		**Lacto**		
**Statistics**	**MEAN**	**SEM**	**MEAN**	**SEM**	**p value**
Other	0.001548314	0.000439488	0.002193821	0.000141268	0.276852368
Bifidobacteriales	0.000042834	4.28E-05	0.002235171	9.36E-04	0.143638118
Coriobacteriales	0.000421716	0.000246127	0.002970328	0.000601906	0.036861121
Bacteroidales	0.7222189	0.056756945	0.6966961	0.018843767	0.704375055
Streptophyta	5.65E-05	5.65E-05	1.74E-05	1.74E-05	0.56717533
Bacillales	0.00E+00	0.00E+00	7.09E-05	4.72E-05	0.272133488
Lactobacillales	0.12120064	0.0333571	0.03286482	0.008295913	0.110345824
Turicibacterales	0.000112891	6.12E-05	0.04865019	3.14E-02	0.262036009
Clostridiales	0.1249025	0.042536811	0.16958349	0.030880544	0.447461283
Erysipelotrichales	0.005403821	0.001992299	0.017476305	0.004893869	0.118251439
Desulfovibrionales	0.0091254	0.007163194	0.006418948	0.000838769	0.742671081
Anaeroplasmatales	1.78E-04	1.26E-04	1.74E-05	1.74E-05	0.329434135
RF39	0.003346736	2.68E-03	0.013073884	5.37E-03	0.20526692
Verrucomicrobiales	0.010683399	0.005327827	0.006605397	0.004579481	0.593398129
