# Supplementary material for: Control of lupus nephritis by changes of gut microbiota
Source: Microbiome. 2017 Jul 11;5:73. doi: 10.1186/s40168-017-0300-8 (PMC5505136; doi:10.1186/s40168-017-0300-8)
Supplement: Supplementary file 3 — Actual abundance numbers and P values for Additional file 1: Figure S3B. (PDF 40 kb) [file 40168_2017_300_MOESM3_ESM.pdf]

Table S2. Actual abundance numbers and P values for Figure S3B.

Weeks	5				
Treatment	casLac	casLac	casPBS	casPBS	
Statistic	MEAN	SEM	MEAN	SEM	p value
Other	1.88E-03	3.27E-04	9.91E-04	4.60E-04	1.71E-01
Bifidobacteriales	7.60E-05	4.46E-05	1.66E-04	9.57E-05	4.40E-01
Coriobacteriales	0.002318456	0.00048959	0.001298869	0.00033862	0.143730833
Bacteroidales	0.65898997	0.07406453	0.72853486	0.04060265	0.45041299
Lactobacillales	0.069507148	0.020791338	0.026217455	0.00328907	0.127583689
Turicibacterales	0.000022	0.000022	0.000274586	0.000274586	0.426012513
Clostridiales	0.20814803	0.06734302	0.07872618	0.02991658	0.15145237
Erysipelotrichales	0.022072799	0.009030085	0.012520727	0.006376837	0.424283897
Desulfovibrionales	0.013110297	0.00475884	0.021730458	0.007876509	0.392460652
Pseudomonadales	0	0	0	0	NA
RF39	0.004098654	0.001851928	0.000746622	0.000610514	0.167676606
Verrucomicrobiales	0.018972559	0.008849053	0.128179226	0.03458968	0.046875547

Weeks	5				
Treatment	Lacto	Lacto	PBS	PBS	
Statistic	MEAN	SEM	MEAN	SEM	p value
Other	0.002133654	0.000440247	0.001780878	0.000994771	0.761473591
Bifidobacteriales	2.17E-02	7.61E-03	2.12E-04	9.49E-05	6.66E-02
Coriobacteriales	1.01E-03	7.92E-05	4.97E-03	2.96E-03	2.73E-01
Bacteroidales	0.54483255	0.05261082	0.74439344	0.09615423	0.13269717
Lactobacillales	0.1259709	0.042995551	0.02562808	0.001771589	0.101682879
Turicibacterales	8.26E-03	4.44E-03	1.04E-04	1.04E-04	1.64E-01
Clostridiales	0.26498701	0.03382335	0.16375219	0.10536628	0.41719608
Erysipelotrichales	0.006740843	0.005929821	0.010262243	0.003974025	0.641765972
Desulfovibrionales	0.001084343	0.001061798	0.01077755	0.007176047	0.270284012
Pseudomonadales	0	0	0.00000885	0.00000885	0.39100222
RF39	0.00630326	0.001338273	0.002672465	0.002555706	0.269150808
Verrucomicrobiales	0.013911013	0.013569701	0.034730827	0.01709158	0.378719185

Weeks	7				
Treatment	casLac	casLac	casPBS	casPBS	
Statistic	MEAN	SEM	MEAN	SEM	p value
Other	1.02E-03	1.12E-04	2.49E-03	9.81E-04	2.32E-01
Bifidobacteriales	1.47E-04	1.17E-04	1.43E-03	7.47E-04	1.85E-01
Coriobacteriales	0.002021423	0.000452472	0.003220117	0.001510557	0.494681854
Bacteroidales	0.74863468	0.05374217	0.75666965	0.0216248	0.89647442
Lactobacillales	0.028693742	0.009577681	0.060462591	0.028803809	0.359493072
Turicibacterales	0.003115383	0.001866299	0.005080427	0.002525604	0.556433601
Clostridiales	0.12458455	0.06431244	0.0974369	0.02744007	0.71733874
Erysipelotrichales	0.016871778	0.000702098	0.033303519	0.010102686	0.202273652
Desulfovibrionales	0.004065542	0.001773447	0.007550715	0.003139972	0.380529794
Pseudomonadales	0	0	0.000013225	0.000013225	0.391002219
RF39	0.001115043	0.000599133	0.001733442	0.001015376	0.622929694
Verrucomicrobiales	0.068505605	0.027813289	0.029443069	0.022818852	0.320771067

Weeks	7				
Treatment	Lacto	Lacto	PBS	PBS	

Statistic	MEAN	SEM	MEAN	SEM	p value
Other	0.001369887	0.000468664	0.002187598	0.000566675	0.310133421
Bifidobacteriales	3.32E-02	1.17E-02	1.72E-03	5.71E-04	7.49E-02
Coriobacteriales	1.55E-03	4.62E-04	3.79E-03	1.61E-03	2.61E-01
Bacteroidales	0.63718011	0.04389722	0.82626518	0.03215729	0.01521871
Lactobacillales	0.099531143	0.012442523	0.04474734	0.009566531	0.014395799
Turicibacterales	5.91E-05	2.30E-05	1.68E-04	9.66E-05	3.46E-01
Clostridiales	0.19652864	0.0320302	0.06156984	0.02446975	0.01709304
Erysipelotrichales	0.011847065	0.002479705	0.042096026	0.007287745	0.020008427
Desulfovibrionales	0.001603372	0.00158781	0.005108926	0.001659496	0.177878762
Pseudomonadales	0	0	0	0	NA
RF39	0.000786616	0.000180855	0.000851832	0.000253996	0.841963996
Verrucomicrobiales	0.015723199	0.004141369	0.011124079	0.008778269	0.658823187

Weeks	9				
Treatment	casLac	casLac	casPBS	casPBS	
Statistic	MEAN	SEM	MEAN	SEM	p value
Other	1.19E-03	1.68E-04	5.05E-03	5.85E-04	4.92E-03
Bifidobacteriales	1.83E-03	1.53E-03	1.82E-03	9.47E-04	9.96E-01
Coriobacteriales	0.003866808	0.001445387	0.005086833	0.001560079	0.587156541
Bacteroidales	0.69708664	0.03703206	0.71183671	0.07224801	0.86377042
Lactobacillales	0.097681943	0.043905201	0.020185638	0.005135462	0.175415564
Turicibacterales	0.005837083	0.001319531	0.006628445	0.001293824	0.683439196
Clostridiales	0.10688213	0.02024616	0.1617455	0.07175755	0.50838603
Erysipelotrichales	0.027706968	0.002257748	0.043127226	0.008271498	0.157952796
Desulfovibrionales	0.002437072	0.000654038	0.012432977	0.006074139	0.198261908
Pseudomonadales	0	0	0	0	NA
RF39	0.002425387	0.001084299	0.003270124	0.000800935	0.555889323
Verrucomicrobiales	0.051970944	0.020233839	0.027943659	0.023111052	0.464327766

Weeks	9				
Treatment	Lacto	Lacto	PBS	PBS	
Statistic	MEAN	SEM	MEAN	SEM	p value
Other	0.001661367	0.000200342	0.002293191	0.001099833	0.609174841
Bifidobacteriales	6.80E-03	4.38E-03	1.11E-03	9.01E-04	2.67E-01
Coriobacteriales	1.70E-03	2.78E-04	5.03E-04	2.71E-04	1.80E-02
Bacteroidales	0.68886328	0.02161542	0.73206258	0.03225564	0.31236925
Lactobacillales	0.097518717	0.026749256	0.068678135	0.013327988	0.373283931
Turicibacterales	2.32E-03	7.65E-04	1.74E-03	1.74E-03	7.75E-01
Clostridiales	0.16846751	0.03422999	0.13423811	0.04456423	0.56471808
Erysipelotrichales	0.006453293	0.001478429	0.014046022	0.005328153	0.251854971
Desulfovibrionales	0.004114528	0.001816422	0.003451929	0.002694578	0.845771309
Pseudomonadales	0	0	0	0	NA
RF39	0.005245345	0.002419961	0.00128824	0.000251619	0.177683736
Verrucomicrobiales	0.015782061	0.002110858	0.039737085	0.013353363	0.17012938

Weeks	11				
Treatment	casLac	casLac	casPBS	casPBS	
Statistic	MEAN	SEM	MEAN	SEM	p value
Other	8.73E-04	7.63E-05	7.86E-03	1.94E-03	3.65E-02

Bifidobacteriales	2.46E-03	4.48E-04	1.69E-03	1.08E-03	5.48E-01
Coriobacteriales	0.013871297	0.005531196	0.007494353	0.002622788	0.352717748
Bacteroidales	0.56173997	0.06310262	0.54480779	0.06213252	0.85467603
Lactobacillales	0.157924052	0.030784302	0.084091084	0.009853121	0.091603386
Turicibacterales	0.038294016	0.012909676	0.022051756	0.013031102	0.409986364
Clostridiales	0.12206408	0.04941929	0.16295402	0.0424685	0.55393208
Erysipelotrichales	0.017164093	0.006764457	0.121427183	0.070927541	0.238006682
Desulfovibrionales	0.0251038	0.016657861	0.003422128	0.001304073	0.28419387
Pseudomonadales	0.000351065	0.000351065	0	0	0.391002219
RF39	0.005269835	0.00294519	0.004433833	0.001526874	0.81212927
Verrucomicrobiales	0.054084167	0.028354038	0.037139677	0.012269051	0.611972554

**Weeks**	**11**				
**Treatment**	**Lacto**	**Lacto**	**PBS**	**PBS**	
**Statistic**	**MEAN**	**SEM**	**MEAN**	**SEM**	**p value**
Other	0.002037516	0.000203155	0.002834064	0.000336026	0.096334406
Bifidobacteriales	6.21E-03	2.83E-03	2.33E-04	1.79E-04	8.81E-02
Coriobacteriales	2.07E-03	5.19E-04	7.00E-04	2.89E-04	5.23E-02
Bacteroidales	0.65827902	0.03168871	0.6820005	0.01541356	0.5224556
Lactobacillales	0.138316054	0.043488781	0.053721392	0.012893275	0.112772349
Turicibacterales	8.59E-03	2.34E-03	1.49E-03	8.76E-04	2.81E-02
Clostridiales	0.15334964	0.04614749	0.22263502	0.02983318	0.243828
Erysipelotrichales	0.008903347	0.002135906	0.013420258	0.005755841	0.504305981
Desulfovibrionales	0.002637979	0.001391379	0.003688562	0.001752111	0.654148595
Pseudomonadales	0	0	0	0	NA
RF39	0.001526645	0.000703586	0.003555801	0.00174738	0.342176794
Verrucomicrobiales	0.017207687	0.007012525	0.014056552	0.006963839	0.758509433

**Weeks**	**14**				
**Treatment**	**casLac**	**casLac**	**casPBS**	**casPBS**	
**Statistic**	**MEAN**	**SEM**	**MEAN**	**SEM**	**p value**
Other	5.08E-03	9.81E-04	1.69E-03	3.38E-04	2.29E-02
Bifidobacteriales	2.35E-03	1.24E-03	3.00E-03	4.78E-04	6.44E-01
Coriobacteriales	0.014413868	0.004567948	0.006526733	0.001132337	0.161304346
Bacteroidales	0.57177803	0.09196368	0.44810113	0.04068063	0.26925288
Lactobacillales	0.154126162	0.055162185	0.040621283	0.007047685	0.108599273
Turicibacterales	0.026008516	0.005100761	0.018255002	0.004513892	0.292450112
Clostridiales	0.06721808	0.0083912	0.13601098	0.0186662	0.02617379
Erysipelotrichales	0.076590135	0.015289918	0.248800936	0.054771049	0.046707685
Desulfovibrionales	0.00956389	0.005383976	0.004718069	0.002382751	0.445040183
Pseudomonadales	2.26282E-05	2.26282E-05	0	0	0.373900966
RF39	0.019271608	0.008772698	0.007935718	0.000403859	0.266050151
Verrucomicrobiales	0.05261331	0.029477267	0.083959808	0.041455005	0.561489629

**Weeks**	**14**				
**Treatment**	**Lacto**	**Lacto**	**PBS**	**PBS**	
**Statistic**	**MEAN**	**SEM**	**MEAN**	**SEM**	**p value**
Other	0.00197856	0.000486302	0.002390608	0.000509941	0.5777235
Bifidobacteriales	1.15E-03	8.55E-04	2.15E-03	1.22E-03	5.24E-01
Coriobacteriales	1.29E-03	6.86E-04	2.67E-03	8.31E-04	2.47E-01
Bacteroidales	0.56671428	0.0657982	0.67993826	0.02499797	0.16751391

Lactobacillales	0.125267816	0.018498725	0.042054841	0.008146301	0.007724948
Turicibacterales	5.90E-03	2.26E-03	9.75E-03	3.29E-03	3.75E-01
Clostridiales	0.18917369	0.04069534	0.1847858	0.03888411	0.94006422
Erysipelotrichales	0.015634245	0.004449733	0.044952811	0.013100892	0.107142535
Desulfovibrionales	0.002331799	0.00213604	0.010617768	0.003457928	0.095258016
Pseudomonadales	0	0	0	0	NA
RF39	0.007996391	0.003095916	0.00290376	0.001450595	0.190465205
Verrucomicrobiales	0.081931813	0.029021054	0.017078962	0.008765417	0.088782908
